# *Clostridium difficile* phages: still difficult?

**DOI:** 10.3389/fmicb.2014.00184

**Published:** 2014-04-28

**Authors:** Katherine R. Hargreaves, Martha R. J. Clokie

**Affiliations:** Department of Infection, Immunity and Inflammation, University of LeicesterLeicester, UK

**Keywords:** *Clostridium difficile*, phage therapy, phage evolution, genomics, lysogeny, gut pathogen, nosocomial

## Abstract

Phages that infect *Clostridium difficile* were first isolated for typing purposes in the 1980s, but their use was short lived. However, the rise of *C. difficile* epidemics over the last decade has triggered a resurgence of interest in using phages to combat this pathogen. Phage therapy is an attractive treatment option for *C. difficile* infection, however, developing suitable phages is challenging. In this review we summarize the difficulties faced by researchers in this field, and we discuss the solutions and strategies used for the development of *C. difficile* phages for use as novel therapeutics. Epidemiological data has highlighted the diversity and distribution of *C. difficile*, and shown that novel strains continue to emerge in clinical settings. In parallel with epidemiological studies, advances in molecular biology have bolstered our understanding of *C. difficile* biology, and our knowledge of phage–host interactions in other bacterial species. These three fields of biology have therefore paved the way for future work on *C. difficile* phages to progress and develop. Benefits of using *C. difficile* phages as therapeutic agents include the fact that they have highly specific interactions with their bacterial hosts. Studies also show that they can reduce bacterial numbers in both *in vitro* and *in vivo* systems. Genetic analysis has revealed the genomic diversity among these phages and provided an insight into their taxonomy and evolution. No strictly virulent *C. difficile* phages have been reported and this contributes to the difficulties with their therapeutic exploitation. Although treatment approaches using the phage-encoded endolysin protein have been explored, the benefits of using “whole-phages” are such that they remain a major research focus. Whilst we don’t envisage working with *C. difficile* phages will be problem-free, sufficient study should inform future strategies to facilitate their development to combat this problematic pathogen.

## *C. difficile* PATHOGENICITY, RIBOTYPES, AND EPIDEMIOLOGY

Over the last few decades the enteric bacterium *Clostridium difficile* has emerged as an important nosocomial pathogen in clinical settings globally, and in particular in Europe, the USA, Canada, and Australia (Kuijper et al., 2006). Despite a general trend in falling case numbers in these countries, *C. difficile* infection (CDI) remains a serious problem. For example there are an estimated 250,000 cases of CDI annually in the USA which result in approximately 14,000 deaths [Centers for Disease Control and Prevention (CDC), 2013]. In addition to the human cost of the disease, the financial costs of treating and managing the infection are significant, with an estimated annual cost of $800 million in the USA and €3000 million in Europe (Bouza, 2012). Number of CDI cases in the UK decreased from 55,498 in 2007 to 14,687 in 2013 (Public Health England, 2013), and this reduction is thought to be attributed to the enormous effort that has been put into CDI (*C. difficile* infection) management strategies such as modified infection control procedures, antibiotic stewardship, and mandatory reporting (Hughes et al., 2013). Therefore it is of concern that despite these efforts, CDI remains a major healthcare challenge.

*Clostridium difficile* infection is generally associated with the production of up to three toxins; toxin A and toxin B, which are encoded on a pathogenicity locus; the PaLoc, and the *C. difficile* binary toxin (CDT; Rupnik et al., 2009). These toxins disrupt the epithelial cell layer of the colon and the resulting inflammatory response contributes to the disease pathology. Symptoms range from mild to serious diarrhea and, less commonly, to the development of pseudomembranous colitis and toxic megacolon which can be fatal (Libby and Bearman, 2009).

Several CDI epidemics have been linked to specific ribotypes such as R027 and R078 (McDonald et al., 2005; Goorhuis et al., 2008), but 100s of different ribotypes have been identified (Wilcox et al., 2012). Ribotyping is a method of assigning strain type based on the amplification of the intergenic region between the 16S and 23S rRNA gene, of which *C. difficile* has multiple copies (O’Neill et al., 1996). The use of next generation sequencing (NGS) technology has revealed the genomic diversity of important ribotypes, such as R027 (Stabler et al., 2009), and one study has mapped the evolution and spread of this ribotype in epidemics across the world highlighting their acquisition of mobile genetic elements and antibiotic resistance genes (He et al., 2012).

The ability of *C. difficile* to form endospores permits its transmission and persistence within clinical settings (Vonberg et al., 2008). In contrast to nosocomial cases, a proportion of patients with CDI acquire *C. difficile* from sources outside the hospital environment (Eyre et al., 2012). The bacterium can colonize individuals asymptomatically, and has reservoirs associated with livestock, food and the natural environment (e.g., Hall and O’Toole, 1935; al Saif and Brazier, 1996; Metcalf et al., 2010, 2011; Zidaric et al., 2010; Pasquale et al., 2011; Hargreaves et al., 2013b). CDI has been suggested to be a zoonotic disease (Rupnik, 2007), and the cross-over of ribotypes between sources has been observed (Janezic et al., 2012) as well as strain transmission between livestock and humans (He et al., 2012). Establishing the source of disease and ecology of this pathogenic species is important for understanding *C. difficile*’s emergence, predominance, and pathology in clinical settings.

## NOVEL WAYS TO COMBAT *C. difficile* INFECTIONS

Current treatment of CDI is with one of three antibiotics: vancomycin, metronidazole, or fidaxomicin; however, treatment failure and recurrent *C. difficile* infection (RCDI) can occur after treatment with any of these antibiotics (Surawicz et al., 2013). The consequences of CDI and its continued clinical prevalence, combined with limited treatment options, have motivated research into alternative therapies to treat infections caused by this bacterium. These include new antibiotics, antimicrobial peptides, bacteriocins, molecular inhibitors such as quorum sensing and riboswitch ligands, toxin targeting molecules such as antibodies, and the use of other bacteria as probiotics or fecal transplants. These approaches are at various stages of development and are discussed in a recent review and are not considered further here [see review by (Zucca et al., 2013)].

Our review focuses on bacteriophage (phage) therapy to treat *C. difficile*. The use of phages as antimicrobials to treat a range of bacterial diseases was developed shortly after their discovery in 1917, and their use as established therapeutics in some countries is well documented (Kutter and Sulakvelidze, 2004). Many reviews have been written on the efficacy and safety of phage therapy (e.g., Abedon et al., 2011; Meaden and Koskella, 2013), and phages for several infectious diseases have had notable successes and are at the stage of clinical trial testing (Brüssow, 2012). Here we aim to review the existing literature on *C. difficile* phages and to highlight the pros and cons, challenges and solutions, associated with developing them as a therapeutic.

## *C. difficile* PHAGE THERAPY

**FIGURE 1:**
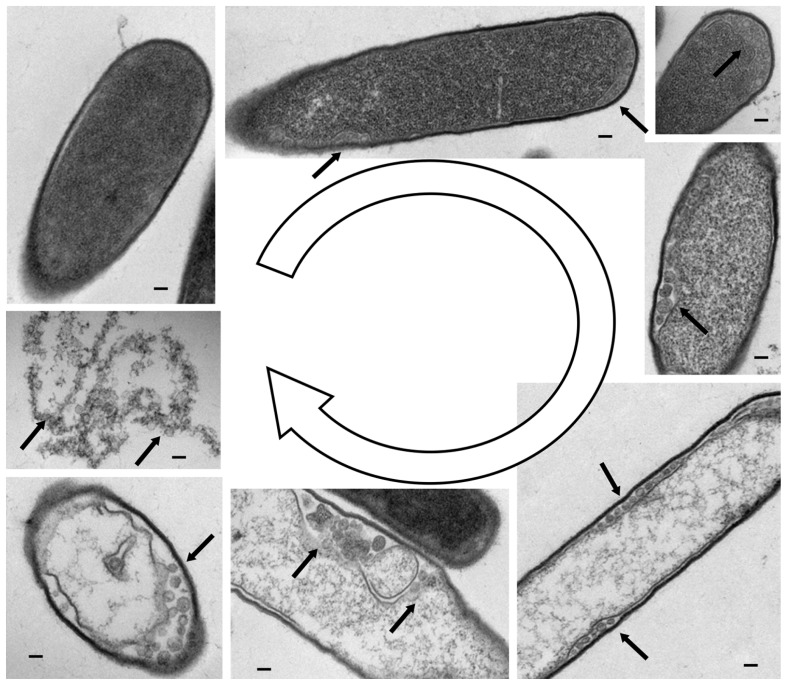
**Lysis of *C. difficile* cells by myovirus phiCDHM1**. Transmission electron microscopy on microtomed samples showing various stages of the phage lytic cycle. Clockwise from top left, a cell with a single attached virion. Photomicrographs show an increasingly granular appearance inside the cell, and the formation of putative capsid structures at the outermost edges of the cell as indicated by arrows. The final image shows the contents of the lysed cell and multiple phage particles. Scale bars represent 10 nm. TEM photomicrographs courtesy of Katherine Hargreaves and Natalie Allcock, the Electron Microscopy Facility, University of Leicester.

The use of *C. difficile* phages as a treatment for CDI would involve orally giving patients a *C. difficile* specific phage preparation. The phages would attach to the receptors on the *C. difficile* cells, and following the phage DNA entering the cell, would undergo replication and ultimately lyse the bacterial cell, releasing phage virions to infect surrounding *C. difficile* cells. This infection and lysis process would be repeated until all the *C. difficile* cells are killed and the infection is cleared. The different stages of a lytic phage infection of *C. difficile* cells are illustrated in **Figure 1**.

There are several reasons why phage therapy would be particularly suited for treatment of CDI. One is that it offers select advantages over existing antibiotic treatments. They include the specific nature of the phage–bacterial interaction, which would avoid exacerbating the gut dysbiosis (disruption of gut microbiota) that can be associated with treatment of CDI (Peterfreund et al., 2012). Another advantage is the ability of phages to replicate in a self-limiting manner at an infection focus. Importantly, the nature and the physical location of CDI may make phage therapy a viable option, as there are likely to be relatively few problems relating to the delivery of phages to the colon. Often patients are infected by a single strain of *C. difficile* (Eyre et al., 2012) and therefore there is not a complex population of organisms to target. In summary, the clinical need to develop new treatments to combat *C. difficile* infection, combined with the problems of antibiotic associated gut dysbiosis, and the physical location and general clonality of the bacterium during infection makes a phage-based therapeutic appealing.

It is perhaps not surprising then that the development of therapeutic *C. difficile* phages has attracted both academic and commercial attention. However, in order for a phage-based therapeutic to be successfully developed for this species, there are several aspects of *C. difficile* bacterial and phage biology that need to be better understood. Considering the global importance of the pathogen, there has been little research on *C. difficile* phages compared to those which infect other pathogenic bacteria, and the information available on phages associated with *C. difficile* has been produced by relatively few research groups. The lack of published work in this area can be seen from a Pubmed search for terms “*C. difficile*” and “phage” which at the time of writing produced 45 results whereas “MRSA” and “phage” resulted in 342 publications. The lack of research in this area likely reflects the technical difficulties of working with anaerobes in general, and *C. difficile* in particular. However, existing research has provided key insights into the host–phage relationship for this species and for the prospects of using phage therapy against CDI.

## SPECIFICITY-RELATED ADVANTAGES OF USING PHAGES FOR TREATMENT OF *C. difficile*

*Clostridium difficile* infection is characterized by a dysbiosis of the human gut microbiota (Manges et al., 2010). This imbalance results in the overgrowth of endogenous *C. difficile* (strains present in the person) or exogenous *C. difficile* (strains acquired from an external source; Eyre et al., 2012). Although mixed infections with multiple strains of *C. difficile* occur, they are thought to do so at a relatively low frequency, with the majority of infections caused by a single strain (Eyre et al., 2012). Healthcare associated epidemics have also been found to be dominated by single types (Loo, 2006; Hubert et al., 2007; He et al., 2012). The limited *C. difficile* strain types in individual patients means that as long as the appropriate phage is delivered, it is likely to be effective in clearing bacteria from infected individuals, and from sets of individuals who are infected during an outbreak setting.

The co-evolution of host bacteria and their predatory phages contributes toward the typically narrow host ranges reported for many phages (Hyman and Abedon, 2010) and it is this precise targeting of hosts that is exploited in the use of therapeutic phages (Kropinski, 2006). The phage mechanism of action is in contrast to the generally wide spectrum of activity that some antibiotics exhibit. This includes vancomycin which is commonly used to treat CDI and concomitantly exacerbates gut dysbiosis (Louie et al., 2012). Phage therapy would take advantage of the highly specific bacterial host ranges often exhibited by phages and therefore *C. difficile* would be removed, but commensal bacteria would be left intact.

Finally, another use of phage therapy would be not to replace antibiotics, but to extend the usefulness of current antibiotics, as the emergence of vancomycin and metronidazole resistance following treatment has been reported (Al-Nassir et al., 2008). Phages could be used as a first line of defense and antibiotics saved for a last resort.

## ADVANTAGES OF *C. difficile* PHAGES TO TREAT BIOFILMS

Unlike antibiotics, phages are self-replicating. Once a susceptible host has been encountered and infected, phages replicate and the delivery of treatment is amplified locally. This quality is particularly desirable when targeting bacterial biofilms as they are notoriously difficult to penetrate and clear with antibiotics (Stewart and Costerton, 2001). Antibiotics have been shown *in vitro* to be ineffectual in clearing *C. difficile* biofilms (Dapa and Unnikrishnan, 2013). This is of clinical significance as *C. difficile* aggregates have been observed on the surface of caecum and colon tissues *in vivo* (Buckley et al., 2011). The successful use of phages to penetrate and disrupt biofilms has been reported in several species, for example in *Pseudomonas aeruginosa* (Hanlon et al., 2001; Fu et al., 2010), *Staphylococcus aureus* (Kelly et al., 2012), and *Campylobacter jejuni* (Siringan et al., 2011). The way in which phages degrade biofilms is often enzymatic with specific phages encoding enzymes that are effective for a target species (Flemming and Wingender, 2010). As *C. difficile* can form biofilms, it is possible that *C. difficile* phages will also have suitable specific enzymatic ability.

## MINIMIZING PROBLEMS WITH PHAGE RESISTANCE

The continuous evolutionary dynamics played out between bacteria and phages has resulted in bacteria gaining multiple and diverse mechanisms to avoid and resist phage infection and predation (Labrie et al., 2010). However, phages have co-evolved alongside their bacterial hosts and have counter-evolved strategies to maintain infectivity in what is often termed an “evolutionary arms race” (Stern and Sorek, 2011). Although evolved resistance to phages has not been reported in *C. difficile*, genome sequencing has revealed the presence of defense mechanisms including a CRISPR/Cas system (Sebaihia et al., 2006), and active type I and type II restriction modification systems (Purdy et al., 2002).

Another way in which bacteria can evolve resistance is to render the phage receptors ineffective. Although no phage receptors have been identified for *C. difficile* phages, one study observed that phages can adsorb onto diverse *C. difficile* isolates, but were unable to lyse them, suggesting that the receptor for the phage used in this instance is well conserved even across isolates that are not susceptible to lytic phage infection (Ramesh et al., 1999). In some Gram-positive bacteria, such as *S. aureus*, the wall teichoic acid has been identified as being essential for phage infection (Xia et al., 2011). Other candidate receptors for *C. difficile* include the S-layer that forms a paracrystaline layer around the whole bacterial cell and is highly variable between strains (Calabi et al., 2001). Although other sugars and proteins protrude through the S-layer, they are present in much lower abundance (Fagan and Fairweather, 2014). The S-layer has also been shown to be a receptor in other species such as the Gram-negative bacterium *Caulobacter crescentus* (Edwards and Smit, 1991).

Although the evolution of resistance to phages for therapeutic purposes is of genuine concern, there are strategies that can be used to minimize selection pressure for bacterial resistance. One such approach is to infect the target organism with a range of phages that have different receptors/modes of infection so changes in several targets would be required for phage resistance to emerge. Importantly, evolving phage resistance may not pose a significant clinical challenge as it can come at a cost to bacterial fitness or virulence (Koskella et al., 2012). This has been observed *in vivo*, in a phage therapy model of *C. jejuni* infections in chickens where emergent phage resistant mutants were not as competitive in growth assays as the non-phage resistant parent strain (Scott et al., 2007). The rates of phage resistance, or how they compare to antibiotic resistance, have not been determined for *C. difficile*, or indeed for many pathogens. However, the impact of phage infection on the development of antibiotic resistance has been investigated in *P. fluorescens* which showed that the application of phages did not accelerate antibiotic resistance (Zhang and Buckling, 2012).

## THE EARLY YEARS OF *C. difficile* PHAGE RESEARCH; PHAGE TYPING

**FIGURE 2:**
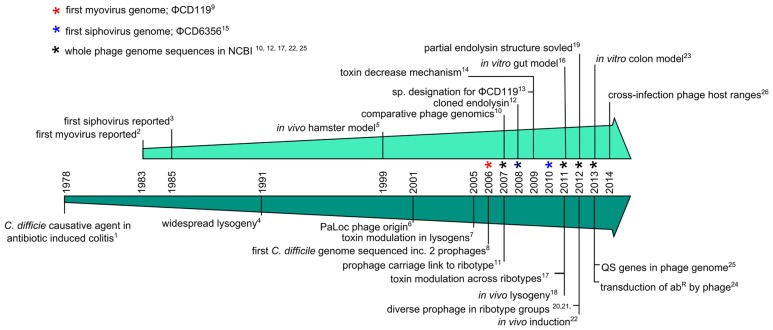
**Timeline charting major events of *C. difficile* phage research**. Milestones are discussed in detail in the text and the references are as follows; (1) George et al., 1978, (2) Sell et al., 1983, (3) Mahony et al., 1985, (4) Nagy and Foldes, 1991, (5) Ramesh et al., 1999, (6) Tan et al., 2001, (7) Goh et al., 2005a, (8) Sebaihia et al., 2006, (9) Govind et al., 2006, (10) Goh et al., 2007, (11) Fortier and Moineau, 2007, (12) Mayer et al., 2008, (13) Lavigne et al., 2009, (14) Govind et al., 2009, (15) Horgan et al., 2010, (16) Meader et al., 2010, (17) Sekulovic et al., 2011, (18) Govind et al., 2011, (19) Mayer et al., 2011, (20) Nale et al., 2012, (21) Shan et al., 2012, (22) Meessen-Pinard et al., 2012, (23) Meader et al., 2013, (24) Goh et al., 2013, (25) Hargreaves et al., 2013c, (26) Sekulovic et al., 2014.

Having discussed the potential benefits of using *C. difficile* phages as a therapeutic, we now discuss research on *C. difficile* phages which has been conducted over the last three decades. They first became the subject of research attention following the realization of the pathogenic nature of the bacterium in the 1970s. **Figure 2** illustrates the progression of *C. difficile* phage research, showing a timeline of significant milestones that followed on from the discovery that *C. difficile* caused pseudomembranous colitis (George et al., 1978).

The lytic activity exhibited by *C. difficile* phages was initially exploited for typing purposes (Sell et al., 1983) and to provide information on the transmission and epidemiology of *C. difficile* strains (Hawkins et al., 1984; Bacon et al., 1988). The carriage of inducible prophages was also investigated in relation to bacterial phenotype and virulence, but did not establish a defined “lysotype” that reflected these phenotypic traits (Nagy and Foldes, 1991). Phage typing was ultimately not successful and its practice was discontinued, this was in part due to their narrow host ranges and strain typing was replaced by emerging molecular typing techniques (Dei, 1989).

## THE EARLY YEARS OF *C. difficile* PHAGE RESEARCH; PHAGE THERAPY

The first *in vivo* model was developed using phage CD140 to treat clindamycin induced CDI in hamsters (Ramesh et al., 1999). This study demonstrated the potential usefulness of phages for CDI, with 14/18 hamsters surviving infection with phage treatment whereas none in the infected control group survived. In the model, a single phage dose of 10^8^ PFU was administered immediately following challenging the hamsters with 10^3^ CFU of *C. difficile*, in all phage treated animals. Two groups then had phage doses administered at 8 hourly intervals up to 48 and 72 h. The longevity of phage therapy protection was tested by re-challenging the surviving hamsters 2 weeks later with a second administration of 10^3^ CFU of *C. difficile*, but no further phage treatment. *C. difficile* and phage counts were obtained from the cecal contents of deceased animals and *C. difficile* numbers in phage treated hamsters ranged from 1 × 10^3^ CFU/g to 3 × 10^6^ CFU/g. Phage were recovered from three of the infected hamsters; two having 10^2^ PFU/g and one with 10^4^ PFU/g recovered. Promisingly, the model showed that phage treatment could protect hamsters against *C. difficile*, that phages could survive in the hamster gut and that *C. difficile* isolates remained susceptible to phage lysis when tested *in vitro*. However, the phage application did not fully clear *C. difficile* and protection was not long lived. This is significant when considering the scope for continued release of *C. difficile* spores from treated patients into the environment and the potential for RCDI episodes, which is highly problematic in antibiotic treated patients (Johnson, 2009).

## A CATALOG OF *C. difficile* PHAGES

The first reported isolation of *C. difficile* phages was in 1983, for the phages described above that were used for typing purposes (Sell et al., 1983). Since then several phages have been described in the literature (Hawkins et al., 1984; Mahony et al., 1985, 1991; Bacon et al., 1988; Ramesh et al., 1999; Goh et al., 2005a, b; Sekulovic et al., 2014). The levels of characterization vary between the studies and include one or several of the following types of data; host range studies, growth dynamics, morphological information, whole genome sequences, and comparative genetics.

The phages from the studies listed above are mainly temperate that were replicated on permissive hosts following either their induction or spontaneous release from a bacterial lysogen. In contrast, some phages were found as “free agents” in sample supernatants. Regardless of their origin, in all cases where the phage genomes have been sequenced, putative integrase genes have been identified which suggests that they can access the lysogenic lifestyle. Indeed, investigations have shown that both environmental and clinical derived *C. difficile* strains carry a diverse and prevalent set of prophages (Nagy and Foldes, 1991; Fortier and Moineau, 2007; Nale et al., 2012; Shan et al., 2012; Hargreaves et al., 2013b; Sekulovic et al., 2014). Collectively, these studies have shown that although some specific prophages appear to be associated with specific ribotypes, for other groups there are high levels of prophage diversity.

## DEVELOPMENT OF ARTIFICIAL PHAGE TREATMENT MODELS

Two recent studies have used a one-phage/strain model in to explore *C. difficile* phage-host interactions in *ex situ* model systems. The first involved studying their dynamics in a batch gut model (Meader et al., 2010) and the second in a multi-vessel model of the colon (Meader et al., 2013). The gut model was performed in batch fermentations in which remedial and prophylactic treatments were tested using ϕCD27 (Meader et al., 2010). Results from both approaches showed significant decreases of CFU, as well as reductions in the levels of toxins A and B relative to no phage treatment. In contrast to the previous hamster model, lower multiplicity of infections (MOIs) were used; 7 in one replicate and 10 in the remaining two replicates. These MOIs resulted in different CFU counts between replicates, and *C. difficile* was cleared from the prophylaxis experiments when an MO1 of 10 was used. PFU counts of ϕCD27 indicated that the phage did not replicate in the model without *C. difficile* present, and there was no reduction in gut bacterial numbers observed based on CFU counts. The effect of metronidazole was tested in the model and found to reduce commensal CFU counts, highlighting the potential to avoid exacerbating the gut dysbiosis by phage treatment over antibiotic therapy.

The second study used a colon model with three vessels to represent the proximal and distal colons (Meader et al., 2013). The phage ϕCD27 was applied daily over 35 days at an MOI of 10. At days 14–21 clindamycin was added to the vessels to produce conditions which would permit *C. difficile* overgrowth. Three replicates were performed, but results were ambiguous. In two of the three, no vegetative *C. difficile* were isolated, but spore counts increased significantly. The third replicate produced CFU and spore counts similar to those of the controls. However, in all three replicates, the toxin levels decreased which is similar to the results in the previous gut model. To determine the cause of the different outcomes between replicates, *C. difficile* recovered from the third were assessed for lysogeny. Nine of ten colonies were positive for ϕCD27 following Mitomycin C treatment and the authors suggest that a lysogen generated early during the experiment went on to predominate the vessel’s *C. difficile* population. What caused this to occur in one replicate and not the others is not known, but could have been due to the composition of the donor gut microbiota. Lastly, the effect on the gut bacterial population was also assessed in this study, expanding on the previous work by using denaturing gradient gel electrophoresis (DGGE) to profile the bacterial community. This was in addition to culture based detection and confirmed through these methods that phage infection did not appear to alter the community structure. Thus, the colon model data illustrates both the potential of phage therapy for CDI to clear *C. difficile*, as well as identifying other factors that could impact phage treatment.

## ISOLATION OF *C. difficile* PHAGES

Phages that infect *C. difficile* have been notoriously difficult to isolate and propagate. Despite the early establishment that multiple phage panels could lyse divergent *C. difficile* strains, studies have reported low frequencies of propagatable *C. difficile* temperate phages on alternative ‘host’ strains via the lytic cycle; 0% (Nale et al., 2012), 3.9% (Sell et al., 1983), 2.1% (Mahony et al., 1985), 3.3% (Mayer et al., 2008), 4.7% (Horgan et al., 2010). This is despite the large numbers of strains (25–94) being screened and used both as sources and as hosts for phages. However, two studies have reported higher rates of propagating phages, with 7.14% (Goh et al., 2005b), and 15.5% (Sekulovic et al., 2014). It is likely that strain relatedness could influence the cross-infection of *C. difficile* phages which may explain low rates of some studies, for example Nale et al. (2012) screened strains only belonging to R027. It is also another motivating factor to seek phages from non-clinical backgrounds.

Two independent research groups have reported unsuccessful attempts to isolate free phages from patient, animal and environmental samples despite the use of multiple hosts and approaches (Goh et al., 2005b; Nale et al., 2012; Shan et al., 2012). A third group used enrichment on 15 *C. difficile* isolates representing eight different ribotype groups, and although they did not find phage in sewage samples (30 samples), they isolated four distinct phages from pooled stool samples (Meessen-Pinard et al., 2012). The researchers went on to show that these were temperate phages which they also detected as prophages in *C. difficile* isolates from the same stool samples.

The difficulties in isolating phages that can propagate on/infect *C. difficile,* have been attributed to its ability to undergo sporulation, a process that may select for lysogenic infections over lytic infections (Goh et al., 2005b). However, virulent phages have been isolated for other spore forming bacteria [e.g., *Bacillus cereus* (El-Arabi et al., 2013)] as well as for other *Clostridium* species [e.g., *Clostridium perfringens* (Volozhantsev et al., 2012)]. In all studies, large scale screenings containing diverse strains appear to be necessary to detect lytically active phages, and in addition to strain relatedness, this may be explained by the high proportion and diversity of prophage carriage between isolates (e.g., Hargreaves et al., 2013b). *C. difficile* lysogens are presumably able to resist both superinfection and secondary infection by related phages (e.g., Goh et al., 2005b). This has implications for the prospects of phage therapy as these lysogens would require therapeutic preparations to encompass a wide enough diversity of phages to counter their presence.

Although virulent phages are considered the most suitable for phage therapy, Fortier and Sekulovic (2013) stated in their recent review that they are not necessarily problem-free as phage therapy candidates for this species, as strains can have numerous prophages and recombination could potentially occur with a virulent phage. Thus, it is both pragmatic and pertinent to work with the phages that exist and which demonstrate lytic activity on specific *C. difficile* strains, but it is clearly advantageous to develop phages for therapy that have minimal or preferably no temperate activity on their target strains.

## INSIGHTS FROM LYSOGENY IN *C. difficile*; IMPACT ON HOST PHYSIOLOGY

Another aspect of phage research in *C. difficile* has been to determine their role in CDI and evolution of this pathogen (e.g., Nale et al., 2012; Hargreaves et al., 2013b). It has been found that several *C. difficile* phages can modulate toxin production during lysogeny (Goh et al., 2005a; Govind et al., 2009; Sekulovic et al., 2011). Further genomic sequencing of isolated phages has described novel phage types (Horgan et al., 2010; Meessen-Pinard et al., 2012) and has revealed surprising genetic features, such as a phage with homologs of the bacterial accessory gene regulatory (Agr) system (Hargreaves et al., 2013c).

Collectively, the research described in this section has significantly expanded our understanding of the potential impact of phage on *C. difficile* physiology and on the suitability for specific phages as therapeutic agents. Clearly when designing phages as a therapeutic product, it is necessary to consider the potential of lysogenic conversion, which is particularly important in *C. difficile* as lysogeny is common. Multiple phage insertion sites have been identified (e.g., Govind et al., 2006; Goh et al., 2007; Williams et al., 2013), and one phage, φCD38-2, does not insert into the bacterial chromosome, but replicates as a circular plasmid (Sekulovic et al., 2011). The instability of both natural and laboratory generated lysogens is well documented, with *C. difficile* cells found to spontaneously release phages (e.g., Mahony et al., 1985), to differentially release their multiple prophages depending on the antibiotic they are exposed to (Sekulovic et al., 2014) as well as following freezing for storage (Goh et al., 2005b). Further to this, Meessen-Pinard et al. (2012) found that artificially generated lysogens produced significantly more phages when exposed to quinolones than the wild-type lysogens did. The impact of sub-inhibitory concentrations of antibiotics on lysogen induction is of relevance as antibiotic treatment may alter the levels of horizontal gene transfer (HGT) in *C. difficile* populations after exposure to quinolones when in patients.

Therefore it is also important to determine how a phage that has a tendency to lysogenize might behave during application as a therapeutic and how it may influence the host bacterium. These effects were examined in the phage, ΦCD119, where lysogens were shown to reduce toxin production (Govind et al., 2009). This occurs via the action of the phage-encoded RepR, which can bind to the promoter regions of *tcdR*, the positive regulator of *tcdA* and *tcdB.* To assess the impact of lysogeny *in vivo* during CDI, the same phage was applied in the Ramesh et al. (1999) hamster model Govind et al. (2011). The results showed that the phage could lysogenize under the conditions present in the mammalian gut, as *C. difficile* isolates were recovered that were unsusceptible to infection with ΦCD119. Prophage integration was confirmed using southern blots and PCR assays. Although lysogeny is not desirable in a therapeutic model, all three of the hamsters treated with phage outlived the controls. The lysogens may have become attenuated as inferred by the decrease in toxin production by the lysogens during culture. However, the toxin levels *in vivo* were not presented and the authors state that they will further determine the attenuation of strains in their future work.

Additionally, studies have shown that the physiological effect of lysogeny varies according to phage or strains used (Goh et al., 2005a; Sekulovic et al., 2011). Goh et al. (2005a) used three phages to lysogenise different strains, and the resulting lysogens produced increased levels of toxin B (3/5 lysogens) and toxin A (1/5 lysogens). Different strains lysogenized with the same phage however, differed in their toxin levels suggesting there is a strain-phage specific interplay that determines toxin levels. Sekulovic et al. (2011) found that two out of five generated φCD38-2 lysogens also stimulated toxin production. Interestingly, there was variation between isolates of the same ribotype. Together, these studies demonstrate that there is considerable variation in the physiological response of phage infection. Further work to establish the underlying mechanisms influencing toxin production in lysogens is needed to fully assess the potential impact it could have on phage therapy in this system.

## INSIGHTS FROM LYSOGENY IN *C. difficile*; HORIZONTAL GENE TRANSFER

In addition to the introduction of novel genetic material in the form of the prophage genome, phage infection also presents the opportunity for generalized transduction to occur. The ability of *C. difficile* phages to mediate horizontal transfer of genetic material in this manner is possible, but has been little studied. While phage induced from a toxigenic strain did not convert a non-toxigenic strain following lysogenisation (Goh et al., 2005b), a recent study demonstrated that another phage, φC2, could mediate the exchange of novel genetic material between *C. difficile* strains (Goh et al., 2013). φC2 was shown to transduce an antibiotic marker *ermB* carried on a transposon 13 kbp in size, after infection with 10^7^–10^8^ PFUml^-^^1^ at MOIs of >0.02 (Goh et al., 2013). The size of the transposon is slightly smaller than the PaLoc which is ~19.7 kbp (Cohen et al., 2000), and the length of DNA that can be transferred remains to be determined. It is known that *C. difficile* phages can access a greater number of strains than revealed by spot test assays alone, as demonstrated by the absorption of phage CD140 to multiple strains without producing lysis (Ramesh et al., 1999). Specific transducing phages could serve as useful molecular tools, as genetic manipulation of *C. difficile* has been difficult to achieve and there are relatively few methods, although a lethal vector system (O’Connor et al., 2006) and the ClosTron system (Heap et al., 2007) are available. Alternatively, phages able to access a broad range of strains without necessarily causing lysis could also be exploited for diagnostic purposes, as similar phage-based detection methods have been developed for other bacterial species such as fluorescently labeled phage, reporter phage or phage amplification assays [see review by (Rees and Dodd, 2006)]. More studies which further characterize phage host ranges are needed in order to assess the impact of transduction between *C. difficile* strains and how this may affect their application.

## *C. difficile* PHAGE HOST RANGES

In general, data on host ranges shows that although some phages can lyse a range of *C. difficile* strains, typically host ranges are restricted to one or a few strains. In the few studies where ribotype information is presented, phages were found to infect across ribotype groups, for example, the phage ΦCD6356 can infect 13/37 strains which belong to five ribotypes (Horgan et al., 2010). The largest host range survey published showed that φCD38-2 could infect 99/207 isolates tested (Sekulovic et al., 2011). Although this seems high, 79 isolates belonged to the same ribotype group and in total only 11 ribotypes were assayed.

The use of phage panels in early typing studies showed that the inclusion of multiple phages resulted in the infection of many of the tested strains by at least one phage (Sell et al., 1983). As these studies predate ribotyping, no information is available about the diversity of the panel of strains being tested (Hawkins et al., 1984; Bacon et al., 1988). A recent study showed that distinct phages induced from different ribotypes, including φCD38-2 could lyse isolates belonging to several ribotypes of human and animal origins (Sekulovic et al., 2014).

The ability of phages to infect a broad range of *C. difficile* strains is considered to be ideal for therapeutic purposes, and this could be achieved by combining phages into a mixture or ‘cocktail.’ To date, only single phages and single *C. difficile* strains have been tested in models for treatment (Ramesh et al., 1999; Meader et al., 2010, 2013; Govind et al., 2011). Extensive host range analysis relating to genetic diversity of both strain and phages is currently being investigated in this laboratory (unpublished). The goal of this work is to determine the interactions between related phages and strains and to develop optimal phage mixtures to treat clinically relevant strains. Identifying phage receptors would help to inform these studies and introduce the possibility of altering phages to target a wider spectrum of strains. The mechanisms of phage resistance in this species also need to be addressed in order to establish the processes constraining the current narrow host ranges that have been reported.

## EFFECT OF *C. difficile* PHAGES ON THE GUT MICROBIOTA

As previously highlighted, a key advantage that phages offer for CDI treatment is their specificity, both to reduce dysbiosis of the gut microbiota associated with CDI and to minimize the potential introduction of virulence genes from the pathogenic *C. difficile* to commensal organisms via transduction or lysogeny. In the gut and colon models, there was no detected impact on gut commensal communities resulting from phage treatment (Meader et al., 2010, 2013). Similarly, lysis of closely and less-closely related bacterial species has been examined in several studies (Sell et al., 1983; Ramesh et al., 1999; Goh et al., 2005b; Horgan et al., 2010). These species include an assortment of clostridial species including *C. sordellii, C. septicum, C. bifermentans*, *C. sphenoides,* and *C. perfringens* with no infective activity reported. However, some cross-infection has been observed as temperate phages from *C. sordellii* were found to be active on *C. difficile* (Schallehn, 1985). In addition, the genetic similarity reported between sequenced phages and other clostridial phages, such as PBSX (Govind et al., 2006), suggests that these phages could share an ancestry or infect across species. The impact *C. difficile* phages have upon patient’s gut microbiota would benefit from the application of NGS technology to investigate the bacterial-phage and phage–phage dynamics during phage treatment. Bacterial and viral genomic and transcriptomic data would be useful for determining the *in situ* population diversity and turnover of phages in order to elucidate the dynamics in this system. Functional data from transcriptomes and proteomes generated from infection models would also provide valuable insight into *C. difficile* phage–host interactions.

## GENOMIC FEATURES OF THE *C. difficile* PHAGES

In this section of the review we discuss the insights into the interactions between *C. difficile* phages and their bacterial hosts gained from the study of their genomes. Many phage genomes are known to contain genes that can alter the physiology of the bacterial cell, including known toxin genes such as those carried by *Escherichia coli* STX phages and the toxin converting *C. botulinum* phage C1 [see review by (Boyd, 2012)]. In order to determine the suitability of specific phages for therapeutic use it is necessary to determine whether they carry genes that could enhance bacterial virulence. Genome analysis can also reveal the genetic relatedness between phages; this information can be used to inform the development of phage cocktails or be useful for other phage applications.

**FIGURE 3:**
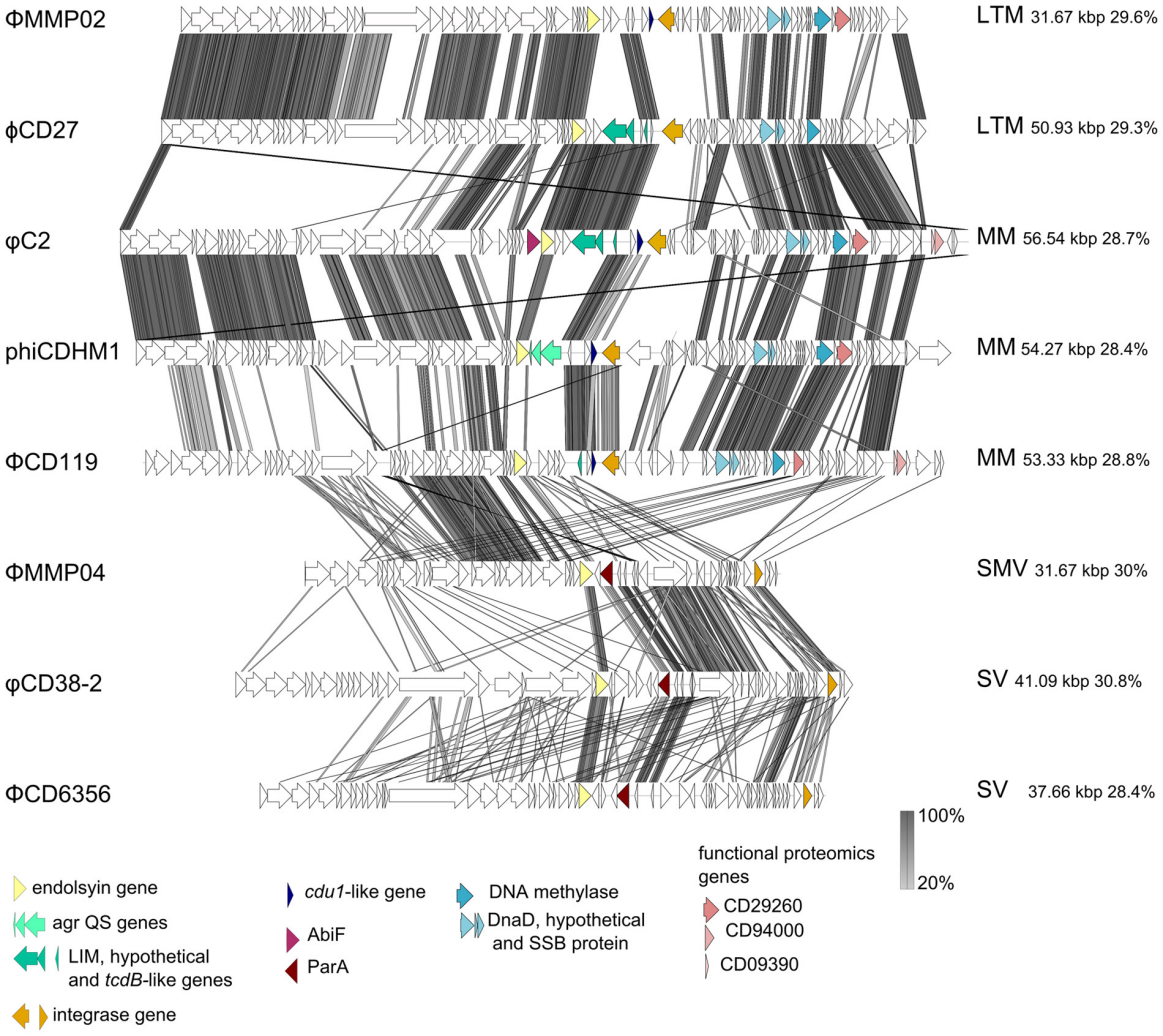
**Sequenced *C. difficile* phage genome similarity and content**. Phage genomes were searched using tblastpx against one another and genome maps were generated using EasyFigv2.1 (Sullivan et al., 2011). The most similar phages are adjacent to one another, phage names are on the left hand side and phage morphological type, genome size and GC content are on the right hand side. The acronyms designate long tailed myovirus (LTM), medium myovirus (MM), small myovirus (SMV), and siphovirus (SV). Accession numbers for each phage genome are as follows: ΦMMP02 NC_019421.1; ϕCD27 NC_011398.1; φC2 NC_009231.1; phiCDHM1 HG531805; ΦCD119 NC_007917.1; ΦMMP04 NC_019422.1; φCD38-2 NC_015568.1; ΦCD6356 NC_015262.1. Specific genes are highlighted to shown their conservation and position between genomes, and are discussed in the text.

All *C. difficile* phage genomes sequenced to date are dsDNA and belong to the Caudovirales (the order of tailed phages). They can be grouped by particle morphology as they differ in size and morphological type which includes the long tailed myoviruses (LTMs) ϕCD27 and ΦMMP04, the medium myoviruses (MMs) ΦCD119, φC2 and phiCDHM1, the small myovirus (SMV) ΦMMP02 and two morphologically distinct siphoviruses (SVs) ΦCD6356 and φCD38-2. In this section we have performed a comparative genome analysis to highlight specific genome features of interest (**Figure 3**). The genome figure has been produced using EasyFig v.2.02 software (Sullivan et al., 2011) and subsequent analysis in Clustal Omega (Sievers et al., 2011).

While no close homologs of the *C. difficile* toxin genes are present in any of the phage genomes, Goh et al. (2007) identified a coding DNA sequence (CDS) in φC2 that has a low level of similarity to *tcdB* at the amino acid (aa) level. This CDS is located within a proposed lysogenic conversion module of the genome, flanked by the lysis and lysogeny control genes and contains CDSs primarily on the antisense strand. This region appears to be present in the other medium and LTMs, but the gene content and exact location of this region varies between phages.

Comparison of this region suggests that recombination between phages may have occurred within this module, as the genes appear to be in cassettes; for example the *tcdB*-like gene of φC2 is adjacent to a putative enzyme (as it contains a DUF955 protein domain, PFam E-value 1.2e-19, with a characteristic H-E-X-X-H motif at aa residues 80–84) and a CDS which encodes a predicted DNA directed RNA polymerase 7 kDa peptide/zinc finger protein [with a LIM domain, (PFam E value 0.066), but not a predicted transmembrane helix as assessed using the TMHMM server v. 2.0 accessed at http://www.cbs.dtu.dk/services/TMHMM/]. This gene cassette is conserved between φC2 and ϕCD27, with each CDS having 100% identity at the aa level between the two phages. The phage ΦMMP02 also encodes a homolog of the third CDS, which shares 100% identity with the genes of φC2 and ϕCD27, but does not have either of the other two CDSs. Also in ΦCD119 we detected an unannotated CDS on the antisense strand which contains a possible LIM protein domain (PFam E-value 0.062), but not the other genes in the cassette, which highlights the high degree of mosaicism between these related phage genomes.

The proposed lysogenic module of phiCDHM1 is notably different to the other sequenced phages. It carries a cassette containing gene homologs of the *agr* quorum sensing system. These genes are not shared with any of the other *C. difficile* phages. They represent a third type of *agr* locus present in relatively few *C. difficile strains* (Hargreaves et al., 2013c).

Another marked feature is the RepR of ΦCD119. Lysogenic infection with ΦCD119 has been found to reduce production of TcdA and TcdB, and RepR appears to repress transcription of *tcdA* and *tcdR* via binding of their promotor regions (Govind et al., 2009). A blastp search of this gene against all the annotated ORFs in each phage genome revealed homologs in phiCDHM1, φC2 and ΦCD6356; phiCDHM1_gp43 shares 30% identity with an E value of 2e-11, the gene phiC2p50 has 35% identity with an E value of 6e-18 and, lastly, ΦCD6356_38 has 27% identity and an E value of 2-10. Whether these genes have homologous functions also remains to be investigated but we can predict that these phages have genes with the capacity to influence cell physiology via regulatory proteins.

A key finding from the study that sequenced the genome of φC2 was that the PaLoc may have had a phage origin due to the sequence similarity between the holin gene and *tcdE* (Goh et al., 2007). This suggestion is further supported in the same study by the identification of a homolog of *cdu1,* Orf 46, which also contains a Penicillinase R protein domain. The CDS is located downstream of the lysogenic conversion module, on the sense strand, and may have a regulatory function. Homologs are present in ΦCD119, phiCDHM1 and ΦMMP02: phiCD119_gp42 has 48% identity and an E value of 1e-20, phiCDHM1 has two homologs; CDHM1_gp40 has 47% identity and an E value of 1e-31 and CDHM1_gp39 has 31% identity and an E value of 5e-19; ΦMMP02 also has two homologs; D863_gp39 has 31% identity and an E value of 2-19 and D863_gp40 has 44% identity and an E value of 5e-27. The functions of these genes are not known nor whether they would be the same for the compared genes.

Lastly, another CDS with a predicted accessory function has been identified in φC2, Orf37, which is a homolog of AbiF (Goh et al., 2007). AbiF is a protein involved in phage abortive infection [see review by Labrie et al. (2010)]. Homologs of this gene are not present in any of the other *C. difficile* phages and while Abi genes are typically encoded by their bacterial hosts, they have been found on a prophage element in *Lactococcus lactis* (Ventura et al., 2007) and in the genome of *L. lactis* phage TP712 (Roces et al., 2013). Although the mechanism by which AbiF performs its function is not fully known (Garvey et al., 1995), the unusual presence of this gene in the genome of phage φC2 suggests it could inhibit secondary phage infection or modulate φC2 replication and may contribute to the low frequencies reported for isolating free phages.

## TAXONOMIC DIVERSITY OF THE *C. difficile* PHAGES

The relatedness of the sequenced phage genomes can be seen in **Figure 3** which includes their sequence similarity at the nucleotide level, resulting from a tblastx analysis performed in EasyFig v.2.0 (Sullivan et al., 2011). The genome sizes vary from ~31 to ~56 kbp, but all share a similar GC content which ranges between ~28–30%. In addition to differences in length, the genomes of the SMVs and the SVs are distinct in terms of their architecture compared to the medium and LTMs. The structural genes are followed by a lysis module, but in the genomes of the SMV and the SVs, the predicted integrases are located following DNA metabolism and DNA replication genes, on the sense strand. However, these phages do have putative lysogenic conversion modules, which are located immediately downstream of the lysis module in each genome. This region encodes predicted proteins with putative accessory functions including membrane or secreted proteins, and all three encode a homolog of ParA, a protein involved in partitioning DNA during cellular division (Bignell and Thomas, 2001). In the genome of ΦMMP04, there is a CDS, D864_gp28, which contains a flagella assembly protein domain, FliH (PFam E value 0.048), as well as a CDS, D864_gp30, which contains a MazE domain (PFam E value 0.025). This region in the two SVs is genetically similar, despite the fact that these phages are of different replication types, *cos* and *pac*, and that the regions containing structural genes are highly divergent to one another.

The subject of taxonomy within *C. difficile* myoviruses was addressed by Lavigne et al. (2009) in a review which predates the publication of either *C. difficile* SV or the SMV genomes. In this review, the authors identified a DNA methylase gene and a DNA replication cassette which are characteristic for the type species ΦCD119. This cassette contains three genes that encode DnaD, a hypothetical protein and a single strand DNA binding protein. The DNA methylase gene and cassette are labeled in **Figure 3**. Although both of the SVs have a gene encoding a predicted single strand DNA binding protein, the other genes are not present in either the SV or SMV genomes and furthermore, these phages do not share 40% of their genes to the type species ΦCD119, or to one another, so could each represent a novel phage type species.

Notably these phage genomes contain divergent regions where genes predicted to encode tail proteins and tail fibers are located, suggesting that these phages could target divergent receptors. There is some similarity between the medium and SMVs in this region of their genomes, and in conserved genes located within it, but there is little to no similarity between the SVs at the aa sequence level. In order to determine whether sequence information can be used to guide phage selection for use in therapeutic cocktails, investigation into the tertiary structures of the putative tail fiber sequences as well as co-absorption assays may be helpful.

## ALTERNATIVES TO THE USE OF “WHOLE PHAGE” TO TREAT CDI

The major disadvantages that have been described against the therapeutic use of *C. difficile* phages are their narrow host ranges and their ability to lysogenize strains of *C. difficile.* To combat these problems, one alternative approach for applying *C. difficile* phage biology in a therapeutic manner has been to clone and express a recombinant version of the endolysin from ϕCD27 (Mayer et al., 2008). Endolysins typically have a cell wall binding domain and an amidase domain, which degrades the bacterial peptidoglycan layer resulting in cell death and lysis. The endolysin has a wider host range than the phage it has been cloned from, which suggests the target of this enzyme in the peptidoglycan structure of different *C. difficile* strains is less variable than that of the phage receptor molecule (Mayer et al., 2008, 2011).

Although this is a non-replicating approach, the endolysin has been cloned into *L. lactis* to demonstrate a proof of principle for delivery to the gut and sites of infection (Mayer et al., 2008). The further investigation of this endolysin found that a truncated version of the N terminus was able to lyse all 32 strains of *C. difficile* tested, and lysed cells more rapidly and effectively than the intact enzyme (Mayer et al., 2011). Surviving cells grew more slowly, and presumably growth rate was subject to the cost of resistance or cell damage. However, the effective host range was also broader, as researchers found that strains of two other clostridial species were lysed by both the intact and partial endolysin, *C. sordellii* and *C. bifermentans*. The endolysin could also lyse less-closely related species, *B. amyloliquefaciens*, *B. cereus*, and *B. subtilis, Listeria innocua, L. monocytogenes serovar 4, L. ivanovii* NCTC 11007; and as the researchers state, this may be explained by the fact that all have a peptidoglycan type of A1γ. How this wider activity of the truncated endolysin impacts upon on the microbial community of the GI tract remains to be tested.

## FUTURE DIRECTIONS

As discussed in this review, there are several potential benefits of using phage therapy to treat patients with CDI. Studies have shown both the efficacy of phages to clear and/or prevent infection and demonstrated the specificity of phages for targeting *C. difficile* in the gut microbial community. However, research into the biology of these phages has also demonstrated a high frequency of lysogeny by known phages and it has highlighted the fact that phages may influence *C. difficile* physiology when present as prophages, or when infecting in the lytic cycle. These different aspects of *C. difficile* phage biology need to be addressed during the development of any therapeutic using such phages. Lysogeny is also undesirable due to the potential transfer of novel genetic material into the recipient cell, and may make the bacterial cell resistant to the phage therapy. This would mean that if the lysogen was spread through a population, the cells that encoded it could not be killed by the same phage that was being given as a therapeutic.

These considerations motivate the continued attempts to isolate strictly virulent phages, but there are also alternative strategies which make use of existing phages. The emerging technology of synthetic biology to alter phage host ranges or to synthesize a strictly lytic phage by mutating a temperate phage is theoretically possible. Such procedures have been achieved for other systems and purposes, e.g., for enhancing antibiotic susceptibility of the host bacterium (Lu and Collins, 2009). Furthermore, synthesizing modified phages has been investigated for a number of pathogenic bacteria such as *E. coli* and *S. aureus* [see review by Moradpour and Ghasemian (2011)]. Genetic manipulation of the phage hosts could also be performed to improve phage isolation or production, as has been done in other bacterial species, for example the removal of prophage elements in *Corynebacterium glutamicum* (Baumgart et al., 2013).

It is worth noting however, that the use of genetically modified phages and indicator hosts could present further difficulties to regulate their use. To avoid the use of GMOs, optimization of existing techniques could be performed, such as continued passage on specific strains to improve host ranges, as has been demonstrated for *P. aeruginosa* (Betts et al., 2013), as well as to devise new approaches, such as using lysogens generated from known phages to screen for less related phages. Additionally, the characterization of host ranges to include more determinant features as suggested by Hyman and Abedon (2010) would aid in understanding the biological parameters in this system.

Also, as yet, no experimental models have included the investigation of multiple phages or strains, and the application of phage cocktails both in *in vitro* and *in vivo* models would help determine their efficacy against a range of *C. difficile* strains. Such experiments could show how resistance could be countered, as well as establish the significance of lysogeny within a multi-phage approach. Such work is currently underway in this laboratory (UoL, 2013). Another option is to expand the models available to assess phage therapy, for example the use of *ex vivo* models such as human epithelial cell lines is being researched in our laboratory (unpublished data, this laboratory) as well as mathematical modeling to assess host range data (unpublished data, this laboratory).

Concurrently, research into how to deliver phages and their production is also required. Phage particles have been found to be inherently stable under specific conditions (e.g., Hargreaves et al., 2013a) and studies of *C. difficile* phages have found they can be stable across a range of pHs and temperatures (Sell et al., 1983; Mayer et al., 2008; Horgan et al., 2010), but stability is variable according to different phages (Mahony et al., 1985), and the use of a bicarbonate buffer administered in an *in vivo* model was necessary for phage viability (Ramesh et al., 1999). Problems with obtaining high titres of phage has also been addressed and researchers have optimized phage production by including divalent cations to infected cultures, altering agar concentrations and by infecting cultures at varying bacterial growth stages which have resulted in phage titres as high as 10^10^ PFU/ml (Mahony et al., 1985; Goh et al., 2005b; Horgan et al., 2010).

Aside from developing the production of a phage therapeutic, further work is needed to establish the consequences of phage infection on both the host bacterium and on the neighboring microbial community. For example RNAseq data could be used to generate transcriptomes, as has been done in other systems, such as in a *Pseudomonas* phage infection model by (Lavigne et al., 2013). In the same study, the authors also utilized proteomic data to probe infection dynamics, highlighting the usefulness of combining several methods of analysis to gain an accurate insight into the temporal dynamics of phage infection. There are few published studies examining the *C. difficile* proteome (e.g., Chen et al., 2013), but they offer valuable data and one such study revealed the presence of phage proteins in CD630 spores (Lawley et al., 2009). Homologs of these genes are highlighted in **Figure 3**, but how phage infection impacts upon sporulation processes is not understood in this species, although it has been well described in another endospore former, *B. anthracis* (Schuch and Fischetti, 2009).

Next generation sequencing has also been applied to aid understanding of the mechanisms facilitating HGT in the normal human microbiota (e.g., Smillie et al., 2011). In *C. difficile*, the PaLoc can be transferred via conjugation as shown during *in vitro* experiments (Brouwer et al., 2013). Understanding the levels of HGT occurring during CDI would be useful in order to determine how this could be impacted upon during phage treatment. It is known that antibiotic treatment can modify the interactions between phage and bacteria (Modi et al., 2013), but this has not been examined for *C. difficile* treatment models including antibody and antibiotic treatment (e.g., Peterfreund et al., 2012) and bacteriocin treatment (Rea et al., 2011). As previously discussed, more information on the bacterial and phage dynamics during CDI would be helpful to assess the impact on prescribing phages as therapeutics.

Research on *C. difficile* phages has revealed key insights into the evolution of this pathogenic bacterium as well as providing resources that can be exploited in many ways. This includes the use of transducing phages, of phages as diagnostic agents, as sources of therapeutic proteins, and indeed as therapeutic agents themselves. To conclude, we concur that *C. difficile* phages are indeed still difficult. They are technically demanding to isolate and propagate and several aspects of their relationship with their bacterial hosts are still unclear. However, their potential value as therapeutics and the emergence of the new sequencing and molecular tools available to researchers should provide answers to the questions which underpin the successful development of a phage-based therapeutic.

## Conflict of Interest Statement

We have strived to write a fair and balanced view of the potential to develop C. difficile phages for therapeutic purposes. The work discussed in this paper was supported by funding from an MRC New Investigator Award to Martha R. J. Clokie G0700855. Martha R. J. Clokie’s laboratory currently receives funding from AmpliPhi Biosciences Corporation, Glen Allen, VA, USA, to facilitate the development of a set of C. difficile phages for therapeutic purposes although data from this work is not included here.

## ABBREVIATIONS

CDI: *Clostridium difficile* infection
CDS: coding DNA sequence
CDT: *Clostridium difficile* binary toxin encoded by two genes *cdtA* and *cdtB*
CRISPR/Cas: a form of RNA based adaptive bacterial immunity to foreign DNA, including phages and plasmids
HGT: horizontal gene transfer
MM, LTM, SMV and SV: abbreviations used to describe the main four morphological types of *C. difficile* phage observed
NGS: next generation sequencing
PaLoc: pathogenecity locus of *C. difficile* containing genes involved in toxicity; *tcdA*, *tcdB*, *tcdE*, *tcdC* and *tcdR*
R027: epidemic ribotype (strain) of *C. difficile*
RCDI: recurrent *C. difficile* infection
SNP: single nucleotide polymorphism
TEM: transmission electron microscopy
TcdA and TcdB: two toxins of *C. difficile* encoded on the PaLoc.
